# Turing instabilities in a mean field model of electrocortical activity

**DOI:** 10.1186/1471-2202-13-S1-P128

**Published:** 2012-07-16

**Authors:** Lennaert van Veen, Kevin Green

**Affiliations:** 1 Faculty of Science, University of Ontario Institute of Technology, Oshawa, Ontario L1H 7K4, Canada

## 

The mean field model formulated by Liley et al. [1] describes the membrane potential and synaptic interaction of excitatory and inhibitory neuron populations in a two-dimensional slab of cortical tissue. When complemented with periodic boundary conditions, it yields a set of fourteen coupled partial differential equations that encompass both highly nonlinear local interaction and long-range interaction through cable equations. We consider these equations as an autonomous dynamical system and aim to parse it using bifurcation analysis. We focus in particular on Turing-type bifurcations from spatially homogeneous equilibria to time-periodic patterns. Such bifurcations have been considered in simplified models but, to the best of our knowledge, not in a physiologically realistic setting [2].

In this study, we fix the model parameters to values that have previously been shown to lead to physiologically interesting γ-range activity [3]. We locate the primary instability when varying the inhibitory-to-inhibitory connection density, known to strongly affect the power spectrum, and the system size.

**Figure 1:**
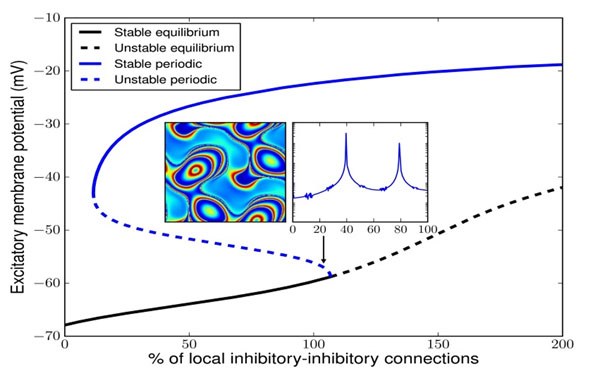
Partial bifurcation diagram of the mean-field model. A periodic solution (blue) bifurcates from an equilibrium (black), both spatially homogeneous. The primary instability, however, is a Turing bifurcation to the pattern in the left inlay. The second inlay shows the power spectrum of the excitatory membrane potential at one point in the domain, with a peak at 40Hz as well as resonance peaks. The periodic solution was computed with 1mm resolution.

Although for small system size, spatially homogeneous periodic solutions appear, for system sizes of a few square centimeters we observe centimeter-scale, oscillating patterns, which appear in subcritical Hopf bifurcations and have a strong γ-band component in their power spectrum. An example is shown in Fig. 1, for a domain of 12.8 by 12.8cm. The black curve denotes stable (solid) and unstable (dashed) equilibria, and the blue curve denotes spatially homogeneous periodic solutions. The latter can be stable to spatially homogeneous perturbations. The primary instability corresponds to a spatial wave number two in both directions. The inlay shows a snapshot of the excitatory membrane potential for the corresponding, mildly unstable, periodic solution near the Hopf bifurcation.

We have implemented a time-stepper in the open-source PETSc environment [4]. It uses a finite difference approximation for the Laplacian and first order implicit time stepping, both for the field equations and for the tangent linear model. The distribution of the grid over CPUs is automated, allowing for efficient parallel computation. This code can be used for the computation of equilibria, traveling waves, periodic orbits, manifolds and other building blocks of dynamical systems analysis, leading to a greater insight in the model's behaviour than could previously be extracted from analysis of spatially homogeneous solutions alone.
